# Higher Hemoglobin A1c Level Is Associated With Poor Outcome of Intracerebral Hemorrhage

**DOI:** 10.3389/fneur.2019.01073

**Published:** 2019-10-10

**Authors:** Huihui Liu, Xia Meng, Chun-Feng Liu, David Wang, Huaguang Zheng, Hao Li, Liping Liu, Yilong Wang, Yongjun Wang, Yuesong Pan

**Affiliations:** ^1^Department of Neurology and Suzhou Clinical Research Center of Neurological Disease, The Second Affiliated Hospital of Soochow University, Suzhou, China; ^2^Department of Neurology, Beijing Tiantan Hospital, Capital Medical University, Beijing, China; ^3^China National Clinical Research Center for Neurological Diseases, Beijing, China; ^4^Barrow Neurological Institute Dignity Health, St. Joseph's Hospital and Medical Center, Phoenix, AZ, United States

**Keywords:** intracerebral hemorrhage, chronic hyperglycemia, hemoglobin A1c, outcome, diabetes mellitus

## Abstract

**Background:** Pre-morbid chronic hyperglycemia is associated with the poor outcome of ischemic stroke, but the association between chronic hyperglycemia, and the long-term outcome of acute intracerebral hemorrhage is still poor understood.

**Methods:** Data on patients with acute intracerebral hemorrhage in the ACROSS-China registry (Abnormal Glucose Regulation in Patients With Acute Stroke Across China) were reviewed. Elevated hemoglobin A1c (HbA1c) level on admission was indicative of chronic hyperglycemia. According to the clinical categories of HbA1c, patients were divided into three groups. Multivariable logistic regression or Cox method was performed to analyze the association of HbA1c and the prognosis of patients with acute intracerebral hemorrhage (poor functional outcome [modified Rankin scale score 3–6] and mortality) at 1 year.

**Results:** A total of 416 patients were included in this study. Fifty-two (12.5%) patients died and 130 (31.8%) had poor functional outcome at 1-year follow-up. The higher levels of HbA1c (≥6.5%) was associated with a poor functional outcome (OR 2.35, 95% CI, 1.28–4.29) and increased mortality (OR 2.63, 95% CI 1.34–5.15), compared with the lowest category. When further stratified by diabetic or non-diabetic medical history, higher HbA1c (≥6.5%) still increased the risk of poor functional outcome (OR 3.42, 95% CI 1.39–8.44) and mortality (OR 4.48, 95% CI 1.64–12.24) in patients with non-diabetic medical history. However, higher HbA1c didn't have the association with the increased risk of poor functional outcome (OR 1.06, 95% CI 0.37–3.03) and mortality (OR 1.20, 95% CI 0.39–3.72) in patients with diabetic medical history.

**Conclusions:** Higher HbA1c was associated with a higher risk of death and poor functional outcome 1 year after intracerebral hemorrhage, especially in patients without a diabetic history.

## Introduction

Diabetes mellitus or hyperglycemia as an independent risk factor for stroke is well-established. A number of studies have demonstrated that either acute or chronic hyperglycemia on admission, is associated with poor prognosis of ischemic stroke, resulting in disability or mortality (1–4). However, evidence on the association of glycemic status and outcomes in patients with intracerebral hemorrhage (ICH) remains limited. Because the pathophysiology of ICH is different from that of ischemic stroke, the association between the blood glucose level, and the outcome of ICH might be different from that of ischemic stroke.

Several researchers have reported the effect of acute glycemic levels on clinical outcomes (5–8). Since acute glycemic status is potentially influenced by the stress response in the setting of an illness *per se*; hemoglobin A1c (HbA1c) level, an indicator of the chronic glycemic state, can more accurately reflect a long-term endogenous exposure to glucose than glucose levels on admission. However, only a few of studies explored the association between HbA1c and the outcome of acute ICH (9, 10). Data from Get with The Guidelines (GWTG)-Stroke registry showed that higher levels of HbA1c (>8.0%) increased the risk of in-hospital death in patients with ICH without a diabetic history; while lower HbA1c (<5.7%) increased the risk of death in patients with or without history of diabetes mellitus (9). However, this study only examined the in-hospital outcomes in ICH, but without a longer term of follow up after discharge.

Therefore, it is unclear whether baseline HbA1c is also associated with long-term outcomes in patients with ICH as well. Hence, we conducted this study to explore the relationship between admission HbA1c and the 1-year prognosis of ICH using a nationwide, multicenter, prospective stroke registry in China.

## Methods

### Study Participants

Data were derived from the ACROSS-China (Abnormal Glucose Regulation in Patients with Acute Stroke across China) registry. The rationale and design of ACROSS-China have been described previously (11). In brief, ACROSS-China registry was a prospective, multicenter, cohort study that consecutively enrolled patients with acute cerebrovascular events, including ischemic stroke, ICH and subarachnoid hemorrhage (SAH) in 35 hospitals across China from 2008 to 2009. The purpose of ACROSS-China intended to investigate the association between abnormal glucose regulation and the outcome of patients after an acute stroke within 14 days from the event onset. The diagnosis of ICH was based on the World Health Organization criteria (12) and combined with brain CT or MRI findings. The study was approved by the ethics committee of Beijing Tiantan Hospital. Informed consent was obtained from all patients who were enrolled before the data collection.

### Data Collection

Demographic, vascular risk factors and baseline clinical data were collected through face-to-face interviews by trained neurologists from participating hospitals within 24 h after admission. Vascular risk factors included a history of hypertension, diabetes mellitus, dyslipidemia, coronary heart disease, atrial fibrillation, heart failure, family history of stroke, tobacco, and alcohol use. Baseline clinical data included National Institutes of Health Stroke Scale (NIHSS), Glasgow coma scale (GCS) score, pre-morbid modified Rankin Scale (mRS) score, systolic blood pressure, and diastolic blood pressure, body mass index (BMI), hematoma location and laboratory values. Hematoma location was divided into lobes, basal ganglia region, brain stem, cerebellum, extension to surrounding spaces. Laboratory values on admission included fasting glucose, HbA1c, high density lipoprotein, triglyceride.

HbA1c was measured within 24 h after admission by using the high performance liquid chromatographicanalysis (HPLC) and in alignment with the Diabetes Control and Complications Trial and National Glycohemoglobin Standardization Program (NGSP) standards (13). Patients were divided into three groups according to their HbA1c levels (<5.7, 5.7–6.4, ≥6.5%) (14–16). In addition, the in-hospital complications of pneumonia and urinary tract infection (UTI) were also included.

### Follow-Up and Outcome Assessment

Patients were followed up for their functional outcome and all-cause mortality at 1 year after the onset of symptom. All follow-up was performed by the trained interviewers who used a standardized protocol through centralized telephone interviews. Poor functional outcome was defined as a score of 3–6 on the mRS (17). Death was confirmed either by the death certificate from the local citizen registry or hospital where the patient was treated.

### Statistical Analysis

The baseline and clinical characteristics were compared between the groups. Continuous variables were presented as mean ± SD or median [interquartile range [IQR]] and categorical variables were presented as frequency (%). ANOVA or the Kruskal–Wallis test was used for continuous variables and the χ^2^ test for categorical variables.

The univariable and multivariable-adjusted odds ratios (ORs) with 95% confidence intervals (CIs) for poor functional outcome were calculated by using a logistic regression model and the univariable and multivariable-adjusted hazard ratios (HRs) with 95% CIs for death were calculated by using a Cox regression model. The lowest clinical categories of HbA1c was used as the reference. Age, sex, and other significant covariates in the univariable analysis were adjusted in the multivariable model. The cumulative probability of death according to the HbA1c categories were assessed by Kaplan–Meier curves and were compared by the log-rank test.

All analyses were conducted with SAS version 9.4 software (SAS Institute Inc., Cary, NC). Two-tailed *p* < 0.05 were considered to be statistically significant.

## Results

A total of 649 patients with ICH were enrolled in this prospective stroke registry. After excluding 233 patients due to missing HbA1c data and lost to follow up, 416 were included in the final analysis (Table 1). Baseline and clinical features of the included and excluded patients were well-balanced except that the HbA1c level of the included participants was higher than those excluded (*p* = 0.002).

**Table 1:** The characteristics of the excluded and included patients.

	**Excluded (*n* = 233)**	**Included (*n* = 416)**	***p-*value**
Age (year), mean (*SD*)	58.9 ± 12.7	58.8 ± 13.2	0.90
Sex (male), *n* (%)	146 (62.7)	268 (64. 9)	0.57
Tobacco use, *n* (%)			0.74
Never	149 (64.0)	255 (61.3)	
Quit	18 (7.7)	38 (9.1)	
Current	66 (28.3)	123 (29.6)	
Body mass index, (kg/m^2^), mean (*SD*)	24.9 ± 4.7	24.5 ± 3.8	0.36
**Medical history**, ***n*** **(%)**			
Diabetes mellitus	20 (8.6)	38 (9.1)	0.81
Hypertension	146 (62.7)	284 (68.3)	0.15
Dyslipidemia	19 (8.2)	18 (4.3)	0.04
Coronary heart disease	18 (7.7)	33 (7.9)	0.93
Atrial fibrillation	3 (1.3)	6 (1.4)	1.00
Heart failure	0 (0.0)	1 (0.3)	1.00
Family history of stroke	18 (8.8)	41 (10.6)	0.48
**On admission status**			
NIHSS score, mean (*SD*)	9.7 ± 8.2	8.7 ± 7.6	0.09
median (IQR)	9.0 (3.0–13.0)	7.0 (2.0–13.0)	
GCS score, mean (*SD*)	13.0 ± 3.0	13.3 ± 2.6	0.35
median (IQR)	15.0 (12.0–15.0)	15.0 (12.0–15.0)	
Pre-morbid mRS	6 (2.6)	5 (1.2)	0.33
score> = 2, *n* (%)			
SBP (mmHg), mean (*SD*)	155.2 ± 24.1	155.1 ± 23.6	0.80
DBP (mmHg), mean (*SD*)	91.5 ± 14.2	90.7 ± 14.1	0.43
Hematoma location, *n* (%)			0.95
Lobes	26 (11.2)	52 (12.5)	
Basal ganglia region	168 (72.1)	300 (72.1)	
Brain stem	16 (6.9)	23 (5.5)	
Cerebellum	13 (5.6)	22 (5.3)	
Extension to surrounding	10 (4.3)	19 (4.6)	
spaces			
**Laboratory values on admission**			
Fasting glucose,	6.3 ± 2.4	6.2 ± 2.1	0.84
(mmol/L), mean (*SD*)			
HbA1c (%), Mean ±*SD*	5.5 ± 1.1	5.8 ± 1.3	0.002
Median (IQR)	5.4 (4.8–5.9)	5.7 (5.2–6.3)	
High density lipoprotein	1.4 ± 0.7	1.3 ± 0.5	0.33
(mmol/l), mean (*SD*)			
Triglyceride (mmol/L),	1.7 ± 1.4	1.8 ± 1.4	0.32
mean (*SD*)			
**Medication in hospital**, ***n*** **(%)**			
Oral hypoglycemic agents	20 (8.6)	38 (9.1)	0.81
Insulin administration	19 (8.2)	31 (7.5)	0.75
Antihypertensive therapy	151 (64.8)	284 (68.3)	0.37
**In-hospital complications** ***n*** **(%)**			
Pneumonia	42 (18.0)	61 (14.7)	0.26
UTI	11 (4.7)	31 (7.5)	0.18

*NIHSS, National Institutes of Health Stroke Scale; SD, standard deviation; IQR, interquartile; SAH, subarachnoid hemorrhage; Q, quartile; GCS, Glasgow coma scale; SBP, systolic blood pressure; DBP, diastolic blood pressure; mRS, modified Rankin scale; HbA1c, hemoglobin A1c; UTI, urine tract infection*.

Among included patients, the average age was 58.8 ± 13.2 years and 64.9% of them were male. The median HbA1c was 5.7% (IQR, 5.2–6.3%). Baseline features of the patients according to the clinical categories of HbA1c were showed in Table 2. Patients with a higher HbA1c levels were more likely to have history of diabetes mellitus, dyslipidemia, higher fasting glucose and triglycerides levels, and be on hypoglycemic therapy. In addition, NIHSS and GCS scores were imbalanced between the groups.

**Table 2:** Baseline characteristics according to HbA1c categories.

**Variable**	**HbA1c <5.7% (*n* = 194)**	**HbA1c 5.7–6.4% (*n* = 129)**	**HbA1c ≥ 6.5% (*n* = 93)**	***p*-Value**
Age (year), mean (SD)	57.5 ± 14.1	59.5 ± 12.9	60.3 ± 11.3	0.15
Male, *n* (%)	121 (63.0)	90 (70.3)	57 (61.3)	0.29
Tobacco use, *n* (%)				0.53
Never	126 (64.9)	74 (57.4)	55 (59.1)	
Quit	16 (8.2)	11 (8.5)	11 (11.8)	
Current	52 (26.8)	44 (34.1)	27 (29.0)	
Alcohol intake, *n* (%)	37 (19.1)	26 (20.2)	12 (12.9)	0.33
Body mass index, (kg/m^2^)	24.1 ± 3.4	24.8 ± 4.2	24.9 ± 3.9	0.24
**Medical history**, ***n*** **(%)**				
Diabetes mellitus	4 (2.1)	7 (5.4)	27 (29.0)	<0.001
Hypertension	122 (62.9)	93 (72.1)	69 (74.2)	0.08
Dyslipidemia	3 (1.5)	7 (5.4)	8 (8.6)	0.02
Coronary heart disease	12 (6.2)	12 (9.3)	9 (9.7)	0.47
Atrial fibrillation	3 (1.5)	2 (1.6)	1 (1.1)	0.94
Heart failure	1 (0.5)	0 (0.0)	0 (0.0)	0.57
Family history of stroke	15 (8.2)	12 (9.8)	14 (17.1)	0.09
**On admission status**				
NIHSS score, median (IQR)	8 (4–15)	5 (2–11)	6 (2–11)	<0.001
GCS score, median (IQR)	14.0 (11.0–15.0)	15.0 (13.0–15.0)	15.0 (13.5–15.0)	<0.001
SBP (mmHg), mean (*SD*)	155.5 ± 23.0	153.2 ± 24.2	157.0 ± 24.2	0.43
DBP (mmHg), mean (*SD*)	90.9 ± 14.4	89.8 ± 13.7	91.4 ± 13.9	0.96
Hematoma location, *n* (%)				0.71
Lobes	24 (12.4)	19 (14.7)	9 (9.7)	
Basal ganglia region	142 (73.2)	91 (70.5)	67 (72.0)	
Brain stem	13 (6.7)	4 (3.1)	6 (6.5)	
Cerebellum	9 (4.6)	7 (5.4)	6 (6.5)	
Extension to surrounding spaces	6 (3.1)	8(6.2)	5 (5.4)	
**Laboratory values on admission**				
Fasting glucose, (mmol/L), mean (*SD*)	5.6 ± 1.2	5.7 ± 1.3	7.9 ± 3.2	<0.001
High density lipoprotein (mmol/l), mean (*SD*)	1.33 ± 0.35	1.32 ± 0.35	1.34 ± 0.74	0.25
Triglyceride (mmol/L), mean (*SD*)	1.60 ± 1.45	1.83 ± 1.22	1.94 ± 1.33	0.02
**Medication in hospital**, ***n*** **(%)**				
Oral hypoglycemic agents	4 (2.1)	6 (4.7)	28 (30.1)	<0.001
Insulin administration	8 (4.1)	5 (3.9)	18 (19.4)	<0.001
Antihypertensive therapy	135 (69.6)	81 (62.8)	68 (73.1)	0.23
Diuretics	17 (8.8)	10 (7.8)	8 (8.6)	0.95
Beta blockers	14 (7.2)	7 (5.4)	5 (5.4)	0.75
**In-hospital complications**, ***n*** **(%)**				
Pneumonia	32 (16.5)	18 (14.0)	11 (11.8)	0.56
UTI	16 (8.2)	7 (5.4)	8 (8.6)	0.57

*SD, standard deviation; NIHSS, National Institutes of Health Stroke Scale; IQR, interquartile; GCS, Glasgow coma scale; mRS, modified Rankin scale; SBP, systolic blood pressure; DBP, diastolic blood pressure; SAH, subarachnoid hemorrhage; HbA1c, hemoglobin A1c; UTI, urine tract infection*.

### Association of HbA1c and Clinical Outcomes

**Figure 1:**
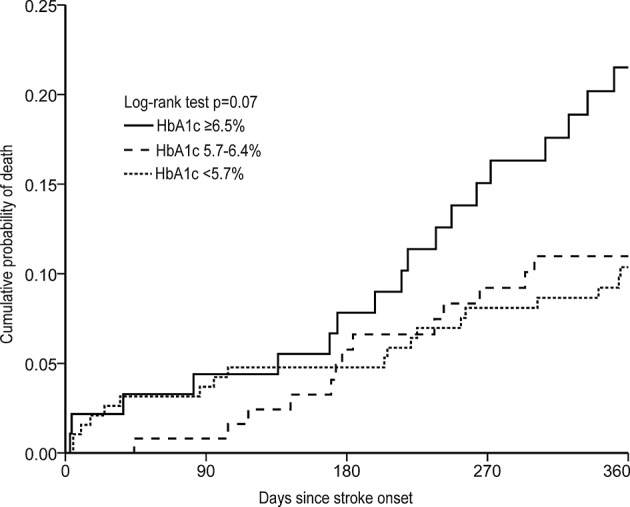
Cumulative probability of death according to HbA1c categories at baseline in patients with intracerebral hemorrhage.

During a 1-year follow-up, a total of 52 (12.5%) patients died and 130 (31.8%) had poor functional outcome. Cumulative probability of death was shown in Figure 1. The association of HbA1c and the clinical outcomes of patients with ICH was demonstrated in Table 3.

**Table 3:** Association between clinical categories of HbA1c and poor outcomes of intracerebral hemorrhage.

**Outcomes**	**HbA1c levels**	***N***	**Events, *n* (%)**	**Unadjusted**		**Adjusted**	
				OR/HR^*^ (95% CI)	***P*-value**	OR/HR^*^ (95% CI)^†^	***p-*value**
^‡^Poor functional outcome	<5.7%	193	59 (30.6)	Ref.	–	Ref.	–
	5.7–6.4%	124	32 (25.8)	0.79 (0.48–1.31)	0.36	1.11 (0.62–2.00)	0.73
	≥6.5%	92	39 (42.4)	1.67 (1.0–2.80)	0.05	2.35 (1.28–4.29)	0.006
Death	<5.7%	194	20 (10.3)	Ref.	–	Ref.	–
	5.7–6.4%	129	13 (10.1)	1.003 (0.50–2.02)	0.99	1.35 (0.64–2.84)	0.43
	≥6.5%	93	19 (20.4)	1.95 (1.03–3.68)	0.04	2.63 (1.34–5.15)	0.005

*OR, Odds ratio; HR, hazard ratio; CI; confidence interval; HbA1c, hemoglobin A1c and Q, quartile*.

* *HR for death, while OR for poor functional outcome*.

† *Adjusted for age, sex, medical history dyslipidemia, NIHSS score, GCS score, triglyceride*.

‡ *Poor functional outcome: modified Rankin Scale score of 3–6*.

Compared with the lowest category of HbA1c, the highest category of HbA1c was associated with the poor functional outcome (OR: 1.67, 95% CI: 1.0–2.80, *p* = 0.05) and increased all-cause mortality (HR: 1.95, 95% CI: 1.03–3.68, *p* = 0.04) (Table 3). After adjustment for age, sex, medical history of dyslipidemia, NIHSS score, GCS score, and triglyceride, the highest category of HbA1c was still associated with the poor functional outcome (adjusted OR: 2.35, 95% CI: 1.28–4.29, *p* = 0.006) and increased all-cause mortality (adjusted HR: 2.63, 95% CI: 1.34–5.15, *p* = 0.005).

When stratified by a positive or negative medical history of diabetes mellitus, the highest clinical categories of HbA1c in patients without history of diabetes mellitus was associated with both poor functional outcome (adjusted OR: 3.42, 95% CI: 1.39–8.44, *p* = 0.008) and increased all-cause mortality (adjusted HR: 4.48, 95% CI: 1.64–12.24, *p* = 0.004) (Table 4). However, we did not find any association between higher clinical categories of HbA1c and poor outcomes in ICH patients with a history of diabetes mellitus.

**Table 4:** Association between clinical categories of HbA1c levels and poor outcomes of intracerebral hemorrhage stratified by diabetic or non-diabetic medical history at 1-year.

**Outcomes**	**HbA1c levels**	**Diabetes mellitus**		**Without diabetes mellitus**	
		OR/HR^*^ (95% CI)	***p-*value**	OR/HR^*^ (95% CI)^†^	***p*-value**
^‡^PoorFunctional outcome	<5.7%	Ref.	–	Ref.	–
	5.7–6.4%	1.33 (0.41–4.37)	0.64	0.85 (0.40–1.78)	0.66
	≥6.5%	1.06 (0.37–3.03)	0.92	3.42 (1.39–8.44)	0.008
Death	<5.7%	Ref.	–	Ref.	–
	5.7–6.4%	1.34 (0.40–4.54)	0.64	1.13 (0.41–3.06)	0.82
	≥6.5%	1.20 (0.39–3.72)	0.75	4.48 (1.64–12.24)	0.004

*OR, Odds ratio; HR, hazard ratio; CI; confidence interval; HbA1c, hemoglobin A1c and Q, quartile*.

* *HR for death, while OR for poor functional outcome*.

† *Adjusted for age, sex, medical history of dyslipidemia, GCS score, NIHSS score, triglyceride*.

‡ *Poor functional outcome: modified Rankin Scale score of 3–6*.

## Discussion

This multicenter cohort study demonstrated that chronic hyperglycemia prior to the onset of ICH was associated with poor functional outcome and increased mortality. This association was significant as HbA1c levels increased. However, when further stratified by medical history with or without diabetes mellitus, we found that chronic hyperglycemia increased the risk of a worsened clinical outcome after the onset of ICH, especially in patients without a diabetic history.

Diabetes mellitus is an established independent risk factor for stroke. The prevalence of previously diagnosed diabetes mellitus in stroke patients was estimated between 10 and 20%, while undiagnosed diabetes mellitus and impaired glucose tolerance accounted for an additional 5–28% (18). A number of studies showed that admission hyperglycemia was a common manifestation in the acute stage of stroke, and always correlated with a poor prognosis and mortality (6, 7, 19). A *post hoc* study of INTERACT2 (Intensive Blood Pressure Reduction in Acute Cerebral Hemorrhage Trial) showed that baseline hyperglycemia was related to 90-days poor clinical outcome following acute ICH (6). A single-center study showed admission hyperglycemia was associated with the increased risk of in-hospital mortality in ICH patients (7). Unlike the blood glucose level on admission, which is influenced by acute stress response, HbA1c represents an extended-period of glycemic level and is a far more reliable and stable test than admission blood glucose level to detect the newly diagnosed diabetes mellitus or the glycemic status in known diabetes mellitus prior to the onset of stroke. Only two cohort studies explored the association between HbA1c and outcome of ICH and found that baseline HbA1c was related with poor outcome (9, 10). However, these two studies only examined in-hospital outcome, but without long-term follow up. Our study added evidence that higher HbA1c was associated with 1-year poor functional outcome and increased mortality. Our finding also indicated that diabetes mellitus itself, rather than the level of HbA1c, predicts poor outcome or death in patients with ICH. Therefore, prevention of diabetes mellitus rather than acute glycemic control is more useful in preventing poor outcome, a finding similar to the results of The Stroke Hyperglycemia Insulin Network Effort (SHINE) trial (20). In addition, patients with known diabetes and normal values of HbA1c had a similar outcome with those with known diabetes and higher values of HbA1c. This phenomenon might be confounded by potential covariants, such as the duration of diabetic state and pre-morbid mRS, which was missing in ACROSS-China study.

HbA1c level reflects the average blood glucose status between 80 and 120 days. The elevated HbA1c is indicative of poor control of hyperglycemia for a period of time. The effect of hyperglycemia on patients with ICH may be related to the exacerbation of hematoma expansion and perihematoma edema (21, 22). This effect may result from the disturbance of osmotic-hemostasis state (23). In animal models, hyperglycemia can cause more profound brain edema and increase neuronal death in areas around the hematoma (24). However, in this study, the data on the volumes of hematoma and perihematoma edema was missing, except that the hematoma location was available, which appeared similar in sizes between groups.

There are several limitations to our study. First, the sample size was a little small. A large-scale study is needed to validate this association. Second, 233 patients were excluded due to missing HbA1c level or no follow ups. Nevertheless, the baseline features between the included patients and those excluded were well-balanced. Third, selection bias might be present because most of patients included in this registry had mild to moderate ICH. Fourth, the population in our study were of Chinese descent; these results may not be generalizable to patients of other ethnicities.

## Conclusions

Our study demonstrated that higher levels of HbA1c at baseline were associated with increased risks of death and poor functional outcome 1 year after ICH, especially significant in patients without a diabetic history.

## Data Availability Statement

The datasets generated for this study will not be made publicly available. Data are available to researchers on request for purposes of reproducing the results or replicating the procedure by directly contacting the corresponding author.

## Ethics Statement

The studies involving human participants were reviewed and approved by the ethics committee of Beijing Tiantan Hospital. The patients/participants provided their written informed consent to participate in this study.

## Author Contributions

YP and HLiu concepted and designed the study. YP and HLi acquired data, analyzed, and interpreted data. HLiu and XM drafted and edited manuscript. C-FL and DW revised and edited the manuscript. YiW, LL, and HZ contributed and designed the study. YoW supported, supervised, and designed the study.

### Conflict of Interest

The authors declare that the research was conducted in the absence of any commercial or financial relationships that could be construed as a potential conflict of interest.
